# Falciparum malaria associated acute kidney injury with polyneuropathy and intra-arterial thrombosis (stroke)

**DOI:** 10.1186/s40001-021-00627-2

**Published:** 2022-01-06

**Authors:** Nausheen Butt, Ejaz Ahmed

**Affiliations:** Sindh Institute of Urology and Transplant, Karachi, Sindh Pakistan

**Keywords:** Malaria, Acute kidney injury, Polyneuropathy, Intra-arterial thrombus

## Abstract

**Background:**

Malaria is still major problem in developing countries, such as Pakistan. Besides fever, body ache and vomiting it can present with acute kidney injury, proteinuria, hematuria and cerebral manifestations which are more common with falciparum malaria. Neurological manifestations are rare presentation of malaria and should be consider in patients who are admitting with features of neuropathy and stroke.

**Case presentation:**

We describe an unusual case of falciparum malaria, complicated by acute kidney injury who developed Polyneuropathy and intra-arterial thrombosis in middle cerebral artery territory. Our patient recovered his renal functions during admission and recovered his power and sensation in his limbs as well after 1 month.

**Conclusion:**

Malaria cause neurological manifestations including axonal and sensory neuropathy, cerebral venous and arterial thrombosis, PMNS, cerebellar signs and symptoms, psychosis, etc. With prompt diagnosis and early treatment they can be cure and regain their motor and sensory functions to normal level.

## Background

In 2019, there were an estimated 229 million cases of malaria worldwide with estimated number of deaths stood at 409,000 [1]. A total of 374,513 confirmed malaria cases have been reported from all the public sector health facilities across Pakistan in annual report of 2019, among which 6.5 million malaria suspects were screened at these health facilities [2]. Falciparum malaria affected 18% patients who diagnosed with malaria in Pakistan according to last surveillance study conducted in 2011 [3]. Besides common presentations there are some uncommon manifestations of falciparum malaria, such as hemiplegia, cranial nerve palsies, myelitis-like syndrome, psychosis, cerebellar ataxia and peripheral neuropathy etc. [4]. We describe an unusual presentation of falciparum malaria with polyneuropathy (both motor and sensory) plus intra-arterial thrombus (causing stroke) in a single young male patient along with acute kidney injury.

## History

An 18-year-old male, resident Sindh, Pakistan, farmer by profession, previously no known co morbids, admitted in Sindh institute of urology and transplantation emergency due to complaints of high grade fever with chills, intermittent, from last 8–9 days, relieve temporarily on taking medicines prescribed by general practitioner nearby, but he was not getting better and developed left flank pain, after 4 days he became drowsy and developed loss of consciousness. No history of seizures, vomiting, loose stools, sore throat, and cough, shortness of breath, hematuria or cola colored urine. He developed decrease urine output for 1 day.

### Antecedents

He got admitted in Nawabshah hospital, where Foleys catheter was passed, 500 ml urine came out in bag, NG (Nasogastric tube) passed and IV line maintained. Given IV fluids. CT scan brain done there which was normal. His routine labs done there showed Hemoglobin (Hb); 8.1 g/dl (normal range 10–12 g/dl), Total Leukocyte Count (TLC): 10.0109/l (normal range 4.0–11.0109/l), Platelets (PLT) 130,000,109/l (normal range 150–400,109/l), Urea 91 mg/dl (normal range 10–40 mg/dl), and Creatinine 3.5 mg/dl (normal range 0.6–1.5 mg/dl). Serum Electrolytes were normal. Liver function tests (LFT’s) were normal, Hepatitis surface antigen (HBsAg) and Hepatitis C antibodies (Anti HCV) were negative. Next day his Urea and Creatinine rise to 160 mg/dl and 4.5 mg/dl, respectively, although his urine output was adequate. He was referred to Sindh Institute of Urology and Transplant (SIUT) hospital Karachi.

### Clinical examination

Here in Emergency, his Glasgow Coma Scale [18] (GCS) was 4/15, Vitals: Pulse 122 b/min, Temperature: 102 F, Respiratory rate 22/min, Blood Pressure: 122/85 mmHg, Pallor + ve, No jaundice, clubbing, edema, no lymphadenopathy. Clinically dehydrated. On examination he had flaccid paralysis of all four limbs (quadriplegia), not moving any limb on painful stimuli. No signs of meningeal syndrome. Reflexes were diminished in all limbs. Planter were equivocal. Rest of systemic examination were unremarkable. No hepatosplenomegaly.

## Biology, imaging and functional examination

### Basic work up showed

Random Blood Sugar (RBS): 182 mg/dl, Oxygen Saturation at room air 93%. Arterial Blood gases (ABG’s): pH 7.4, PCO_2_ 33.0, PO_2_ 95 and HCO_3_ 24. *Hematology* Hb: 6.2 g/L, TLC 14 × 10^9^ L, platelets 73 × 10^9^ L. Prothrombin time (PT) 13.5, INR: 1.28 *BIOCHEMISTRY*: Urea 261 mg/dl, Creatinine 3.5 mg/dl, Sodium (Na): 177 mmol/l (normal range 135–145 mmol/l), Potassium (K) 4.5 mmol/l (normal range 3.5–5.0 mmol/l), Chloride (Cl) 140 mmol/l (normal range 100–106 mmol/l), and Bicarbonate (HCO_3_) 29 mmol/l (normal range 24–30 mmol/l). Calcium (Ca) 6.4 mg/dl (normal range 8.5–10.5 mg/dl), Albumin 2.44 g/dl (normal range 3.5–5.0), and Phosphorus (PO_4_):3.65 mg/dl (normal range 2.7–4.5 mg/dl), Liver function tests (LFT’s) were normal except gamma GT 137 IU/l (normal 9–50 IU/l), alkaline phosphatase 275 IU/l (normal up to 104 IU/l). Serum Lactate dehydrogenase (LDH): 1194 IU/l (normal 45–90 IU/l). Urine output was 900 ml in urinary bag. *Malarial parasite (MP)* thick and thin films showed positive with plasmodium falciparum. *Urine detail report (Urine DR)* showed active urine sediments, i.e., RBC/s Numerous, Protein + 3.

He was tested negative for COVID-19 PCR (nasal swab sample).

Lumbar puncture (LP) was performed and Cerebral Spinal Fluid (CSF) sent for analysis which showed Glucose 117, Protein 97, LDH 80 and TLC count of 20 with 45% Neutrophils and 55% lymphocytes. Culture negative came out later after 2 days. *Urinary chemistry* Sodium Na: 33 mmol/l, K 35 mmol/l, and Cl 31 mmol/l. Fractional excretion of sodium was 1%. He was having free water excretion with good urine output of around 2 l/day.

Spot Urine Protein 286 mg/dl, urine creatinine 52 mg/dl, P/C ration of 5.5 g. Complete Serology was ordered including C3, C4, ANA profile, serum IgA, IgG, IgM, ANCA titer and ASO titer. Antiphospholipid antibodies (APLA) serology and thrombophilia profile (factor v, protein C, S and anti-thrombin 3) were also sent.

### Clinical and biological evaluation

Neurologist was consulted who ordered MRI Brain and EMG/NCS After 3 days his urea and creatinine were 135 mg/l and 1.51 mg/dl, respectively, Na: 164 mmol/l, K: 3.4 mmol/l, Cl: 133 mmol/l and HCO_3_ 22 mmol/l. Therefore, he was having free water excretion which was corrected by giving free water from NG. After 2 days, he became conscious, awake alert with GCS of 15/15*

#### MRI and MRA findings

Small acute bilateral parietal infarcts and thrombosed intracerebral carotid artery. Pan sinusitis with bilateral mastoiditis. Arrows in these pics suggesting the ischemic areas and sites of thrombosis in vessels.
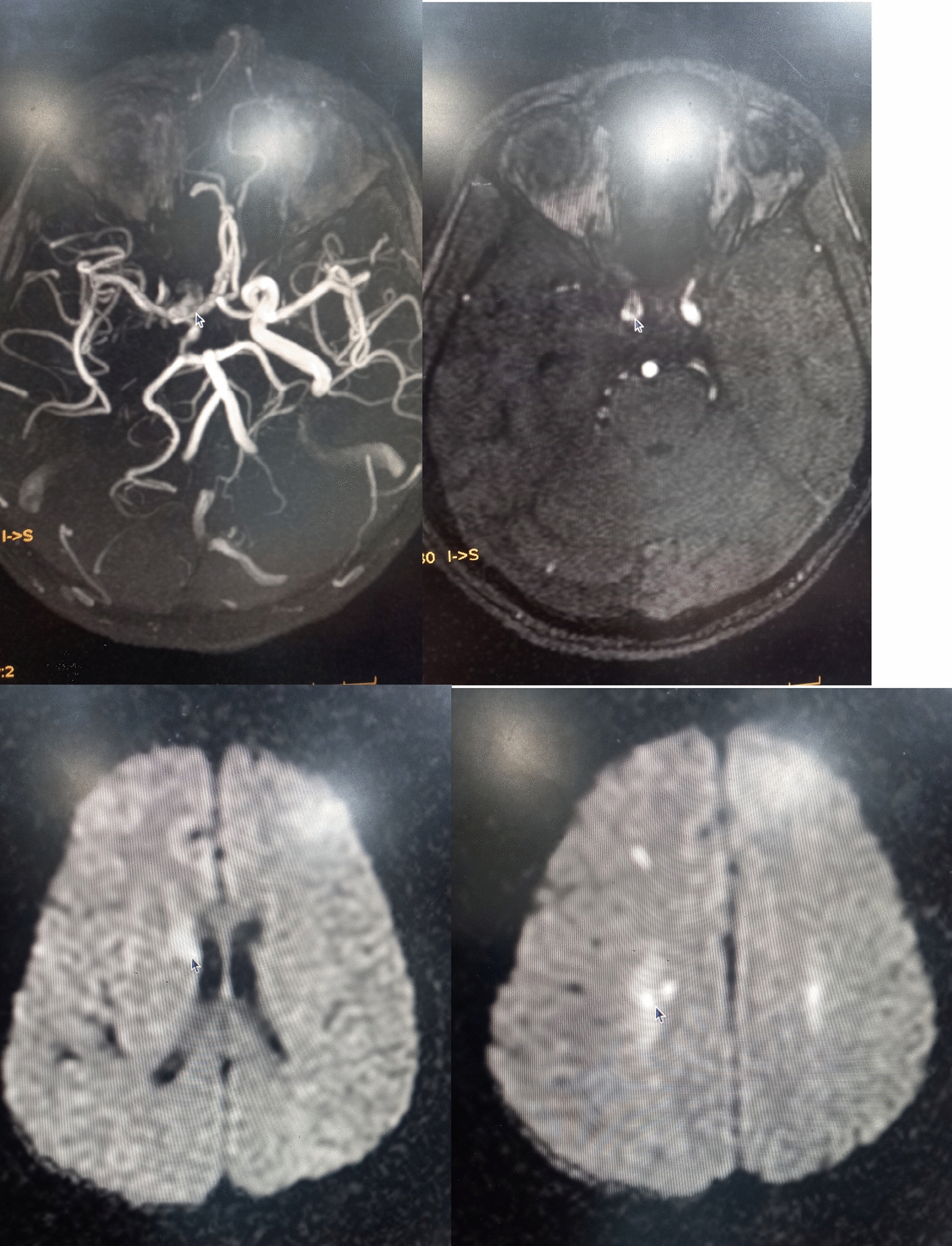


### EMG/NCS

Bilateral median (APB), post tibial (AH) and right ulnar (ADQ) motor nerves conduction study normal. Bilateral Peroneal (EDB) and left ulnar (ADQ) motor nerves conduction studies showed low amplitude and non-recordable F wave latencies. Bilateral H reflex studies were normal. Bilateral median F2 Ulnar F5 and Radial sensory nerves conduction studies were normal. Bilateral Sural sensory nerve conduction studies were non recordable. So that showed sensory motor axonal polyneuropathy more in lower limbs.

### Diagnosis

Severe malaria with gravitational signs, pre renal form of acute renal failure, intracellular dehydration, sensory-motor polyneuropathy, cerebral artery thrombus.

### Treatment and hemodialysis

He was initially started on Intravenous fluid, started on broad spectrum antibiotics and had one session of Hemodialysis of 2 h duration, without ultrafiltration and without heparin. He was started on anti-malarial IV Artesunate 120 mg four doses 12 h apart then IV OD for 5 days more then shifted to Tab. Artemether/Lumefantrine (40 mg/240 mg) PO BD for 2 days. He was given IVF 1/2 strength saline 1 l 8 hourly plus symptomatic management. Next day (after 1 session of Hemodialysis) his urea 145 mg/dl, Creatinine 2.56 mg/dl, Calcium 7.57 mg/dl, Albumin 3.1 g/dl, Na: 160 mEq/l, K: 3.8 mEq/l, Cl 125 mEq/l, HCO_3_ 27 mEq/l, Phosphorus: 7.25 mg/dl. After making final diagnosis He was given antiepileptic prophylactically Levetiracetam 250 mg BD, vitamin B6 and vitamin B12, for thrombosis we started inj. Clexane 60 mg (ENOXAPARIN SODIUM) Subcutaneous (SC) OD. After further 4 days his urea and creatinine became normal 46 mg/dl and 0.49 mg/dl, respectively, and Na became 148 mmol/l. He started moving his right arm, power improved in upper limbs but not in lower limbs. Inj. Clexane continued for 2 weeks more. His Na became 140 mmol/l after further 4–5 days and Urine output became normal and free water excretion decreased. His power improved gradually in all four limbs. He started walking with support. On discharge Power in upper limbs were 5/5 and lower limbs 4/5 in Right and 3/5 in left lower limb proximally and 4/5 distally with intact reflexes and sensation. Autoimmune serology was negative C3, C4, ANA, Anti Ds DNA). Later his thrombophilia profile (Protein C, S, anti-thrombin 3 and factor V mutan, homocysteine levels) came normal and APLA (anti phospholipid antibodies) negative. In addition, after 1 month on follow-up in OPD he had normal power in all four limbs.

## Discussion and conclusion

Classical presentation of falciparum malaria is seen in 50–70% of the cases with the rest having atypical manifestations.

### Malaria and kidney

Glomerunephritis is fairly common in falciparum malaria with albuminuria is seen in 20–50% of cases and acute diffuse malarial nephritis is rare with hypertension, albuminuria and edema. We also see Nephrotic syndrome as immune complex mediated nephropathy develops weeks after the malaria, it may be progressive and may require treatment with steroids or immunosuppressant [5]. More recent studies have also found IgA deposits in malaria [29, 30], Eosinophilic glomerulonephritis [31]. Autoantibodies have also been detected in patients with malaria-associated glomerulonephritis, but its role has yet to be determined [32]. Hemolytic–uremic syndrome (HUS) is also described in malaria [33]. In malaria–endemic regions, acute kidney injury (AKI) can occur in up to 40% of adult patients and it is associated 75% mortality when renal replacement therapy (RRT) is not started in time [6]. Microcirculation disorders, anoxia and subsequent necrosis of the glomeruli and renal tubules are responsible for this serious complication. ATN occurs in 1–4% of cases of *P. falciparum* infection. Disseminated intravascular coagulation also may cause or aggravate this problem. Histologic findings of malaria pigment in distal convoluted tubules are consistent with the reports of intravascular hemolysis and increased cell-free hemoglobin and heme in malaria-associated AKI [27, 28].

### Neuropathy during malaria: literature and diagnostic steps

In literature various neuropsychiatric manifestations of falciparum malaria in different case reports and case series [7–19] have been described which are classified mainly into three groups [14]: (1) Sequelae of cerebral malaria, e.g., hemiplegia, cranial nerve palsies, neuropsychiatric signs and symptoms, such as psychosis, (2) Neuropsychiatric symptoms during acute stage as a presenting illness. (3) Neurological or psychiatric sign occurring with a latency of several days to weeks (generally within 2 months) after an episode of successfully treated *P. falciparum* malaria [12]. Most of the times, these neurological manifestations disappear after successful treatment of malaria itself with residual symptoms which disappear over the course of time with symptomatic management. Among various neuropsychiatric manifestations/sequelae of malaria GBS, such as polyneuropathy [17], acute and delayed cerebellar ataxia [24], acute inflammatory demyelinating polyneuropathy AIDP [16] autonomic neuropathy [15], bilateral axonal polyneuropathy [18] are reported mainly from Indian subcontinent. Exact cause of polyneuropathy is not known but has been attributed to immune mediated capillary damage, toxic oxygen radicals, tumor necrosis factor, parasitic emboli obstructing the vasa nervorum, neurotoxin release, nutritional and metabolic disturbances [16]. Most of PMNS characteristics can fit into the diagnosis of postinfectious encephalitis, ADEM (acute diffuse encephalomyelitis) or AIE (autoimmune encephalitis). Literature review of previous case reports showed that MRI is best modality to diagnose cerebral manifestations and CT failed to reveal any acute abnormalities. MRI abnormalities consisted of increased signal uptake in various regions, including the periventricular areas, pons, thalamus, corona radiata, internal capsule, and cerebellum. CSF analysis was performed in those cases, revealing a lymphocytic pleocytosis (> 5 lymphocytes, range 10–76) in 50% and elevated protein in 69% of cases [19]. Our patient had same features, normal CT scan with positive findings in MRI and lymphocytic predominance in CSF with high protein. Cerebral malaria may be associated with raised intracranial pressure due to cerebral edema.

### Arterial thrombus during malaria-framework of an infectious vasculitis

Infections including several viruses, fungi, bacteria, and parasites are associated with stroke. In patients with infective endocarditis, septic cerebral embolism is a significant risk factor for embolic strokes. Evidence also suggests that systemic infection may trigger acute stroke in patients with vascular risk factors.

Falciparum malaria infection influences blood coagulation by various interacting path biological mechanisms, the most important being the overwhelming response of the host to sepsis resulting in a cytokine storm. In addition, the parasite infects the red cells leading to changes in the red cell phospholipid composition which supports blood coagulation. Monocyte–macrophage system also gets activated in this infection compounding the hypercoagulable state. Heavy parasitemia leading to occlusion of hepatic microcirculation leads to abnormalities in synthesis and secretion of coagulation factors and their inhibitors. A hypercoagulable state resulting from severe malaria may be responsible for this rare and potentially fatal complication. Previous studies showed that ADAMTS13-activity was found to be reduced in malaria, indicating that a decrease in this enzyme probably occurs at later stages of malaria [21] and decreased blood platelet counts and elevated levels of vWF at very early stages of malaria infection [22]. One study showed that the thrombin generation potential might have a role in advanced infections and the development of complications during a *P. falciparum* infection [23]. Cerebral venous sinus thrombosis has been reported in previous case reports to be associated with severe malaria [20, 35]. There is case report showing pulmonary embolism associated with falciparum malaria too [34]. Malaria with stroke was reported in 1999 with lesion in the region of the middle cerebral artery without any associated encephalitis [36]. Later in 2008 a case report of vertebrobasilar stroke with falciparum malaria was published as well presenting predominantly with signs of lateral medullary and cerebellar infarctions [37].

Intra-arterial thrombosis which is very rare presentation especially at this young age without any risk factors, such as Hypertension, Diabetes, dyslipidemia, smoking and medical history, and we excluded COVID-19 in this patient as well. The development of neurological manifestation after diagnosing malaria and improvement after starting antimalarial therapy strongly suggest that the ischemic stroke was mainly due to falciparum malaria. There was no atherogenetic changes in carotid arteries as suggested by normal carotid Doppler and normal fundoscopy. Our case is unique in the way that stroke have never reported with polyneuropathy in a single patient yet. Although autonomic neuropathy and axonal neuropathy were reported in various case reports in past separately but our case has both sensory and axonal motor neuropathy in one patient. Plus our patient’s neurological manifestations became better gradually with full recovery within 1 month after treatment. Our patient didn’t receive steroids.

## Data Availability

Contact corresponding author for detailed reports and data.
